# Expression of a Mutant SEMA3A Protein with Diminished Signalling Capacity Does Not Alter ALS-Related Motor Decline, or Confer Changes in NMJ Plasticity after BotoxA-Induced Paralysis of Male Gastrocnemic Muscle

**DOI:** 10.1371/journal.pone.0170314

**Published:** 2017-01-19

**Authors:** Elizabeth B. Moloney, Barbara Hobo, Fred De Winter, Joost Verhaagen

**Affiliations:** 1 Department of Regeneration of Sensorimotor Systems, Netherlands Institute for Neuroscience, an Institute of the Royal Netherlands Academy of Arts and Science, Amsterdam, The Netherlands; 2 Department of Neurosurgery, Leiden University Medical Centre, Leiden, The Netherlands; 3 Centre for Neurogenomics and Cognitive Research, Vrije Universiteit Amsterdam, Amsterdam, The Netherlands; University of Queensland, AUSTRALIA

## Abstract

Terminal Schwann cells (TSCs) are specialized cells that envelop the motor nerve terminal, and play a role in the maintenance and regeneration of neuromuscular junctions (NMJs). The chemorepulsive protein semaphorin 3A (SEMA3A) is selectively up-regulated in TSCs on fast-fatigable muscle fibers following experimental denervation of the muscle (BotoxA-induced paralysis or crush injury to the sciatic nerve) or in the motor neuron disease amyotrophic lateral sclerosis (ALS). Re-expression of SEMA3A in this subset of TSCs is thought to play a role in the selective plasticity of nerve terminals as observed in ALS and following BotoxA-induced paralysis. Using a mouse model expressing a mutant SEMA3A with diminished signaling capacity, we studied the influence of SEMA3A signaling at the NMJ with two denervation paradigms; a motor neuron disease model (the G93A-hSOD1 ALS mouse line) and an injury model (BotoxA-induced paralysis). ALS mice that either expressed 1 or 2 mutant SEMA3A alleles demonstrated no difference in ALS-induced decline in motor behavior. We also investigated the effects of BotoxA-induced paralysis on the sprouting capacity of NMJs in the K108N-SEMA3A mutant mouse, and observed no change in the differential neuronal plasticity found at NMJs on fast-fatigable or slow muscle fibers due to the presence of the SEMA3A mutant protein. Our data may be explained by the residual repulsive activity of the mutant SEMA3A, or it may imply that SEMA3A alone is not a key component of the molecular signature affecting NMJ plasticity in ALS or BotoxA-induced paralysis. Interestingly, we did observe a sex difference in motor neuron sprouting behavior after BotoxA-induced paralysis in WT mice which we speculate may be an important factor in the sex dimorphic differences seen in ALS.

## Introduction

The development, maintenance and regeneration of neuromuscular junctions (NMJs) is highly dependent on the function of terminal Schwann cells (TSCs), specialized cells that envelop the motor nerve terminal [1–3]. Semaphorin 3A (SEMA3A) expression is up-regulated in TSCs at the NMJ following experimental denervation of the muscle (BotoxA-induced paralysis or crush injury to the sciatic nerve) or in the motor neuron disease amyotrophic lateral sclerosis [4]. This expression is selective to the TSCs which envelop the NMJ of fast-fatigable (TypeIIb) muscle fibers. The TypeIIb fibers are the first to undergo denervation in ALS, and show limited plasticity in sprouting and forming functional ectopic synapses upon denervation [5–9]. Re-expression of SEMA3A at the NMJs of TypeIIb fibers is thought to play a role in the selective degeneration of these junctions by creating a growth-inhibitory environment around the motor nerve terminal.

During development, SEMA3A is vital for normal patterning and growth of nerves, bones and heart [10], as well as vascular patterning [11]. The role of SEMA3A in motor axon pathfinding has been studied in detail: SEMA3A signaling is involved in the establishment of functional motor circuits and spinal motor connections [12] and dorsal motor axon extension [13]. However, its role upon re-expression at the NMJ after denervation is not yet clear, but it may influence the selective plasticity of nerve terminals after injury or in disease.

SEMA3A signals via a membrane receptor complex composed of a ligand binding component (neuropilin-1; NRP1) and a signal-transducing component (plexin-A; PLXN-A) [14]. The downstream elements of the SEMA3A-signaling pathway, the collapsin response mediated proteins (CRMPs) mediate cytoskeletal changes that govern growth cone collapse and repulsion during development [15]. In the mature nervous system, abnormalities in axon-guidance molecule signaling may lead to impaired neuronal connectivity [16,17], a common feature in the presymptomatic stages of many neurodegenerative diseases. In fact, members of the SEMA3-signaling pathway have been linked to the progression of various neuropathological diseases such as Alzheimer’s disease [18–21], Parkinson’s disease [22] and ALS [23–26]. Direct manipulation of the SEMA3A-NRP1 signaling pathway in ALS mice, either by motor neuron specific knockout of NRP1 [27], or by using an anti-NRP1 antibody to disrupt SEMA3A binding [26] leads to improved motor function in the diseased mice. In the current study we sought to understand the role of SEMA3A in regulating NMJ plasticity by diminishing the chemorepulsive abilities of the protein at the synapse in two denervation paradigms: in ALS mice, and in BotoxA-induced paralysis of wild-type mouse gastrocnemic muscle.

The K108N-SEMA3A mutant mouse expresses a variant of the SEMA3A protein which maintains its ability to bind to NRP1 but is no longer able to interact with the signal transducing components of the receptor complex, the PLXNs. This SEMA3A mutant has lost approximately 85% of its repulsive activity [28]. We cross bred the K108N-SEMA3A mutant mouse with the G93A-hSOD1 ALS mouse to look at the effects of diminished SEMA3A signaling on the ALS phenotype. ALS mice that either expressed 1 or 2 mutant SEMA3A alleles demonstrated no difference in ALS-induced decline in motor behavior. We also investigated the effects of BotoxA-induced paralysis on the sprouting capacity of NMJs in the K108N-SEMA3A mutant mouse, and observed no change in the differential neuronal plasticity found at NMJs on fast-fatigable or slow muscle fibers due to the presence of the SEMA3A mutant protein.

## Materials and Methods

### Experimental animals

Male mice, heterozygous for the K108N mutation in the SEMA3A gene [Line 808 in the C57BL/6J background [28]], were kindly provided by Dr. Alex Kolodkin and Dr. David Ginty (The Johns Hopkins University School of Medicine, Baltimore, MD). The line was maintained as heterozygous through breeding with wild-type littermate females or imported females of identical background (C57BL/6J, Harlan, The Netherlands). These mice formed the basis for the Botulinum Toxin A (BotoxA)-induced paralysis experiment and the behavioral experiment as outlined below.

To generate animals for the BotoxA experiment, a heterozygote K108N-SEMA3A line was created by cross-breeding heterozygote males and wild-type female littermates. Of the resulting offspring, heterozygote individuals were cross-bred together in order to produce litters containing the three genotypes required for the experiments listed below: wild-type (WT, -/-), heterozygote (K108N-SEMA3A Het, N/-) and homozygote (K108N-SEMA3A Hom, N/N). Ear snips for genotyping were taken at the time of weaning (~21 days of age) and enabled identification of animals during the experiment.

For the behavioral experiment, we introduced the K108N-SEMA3A mutation into the G93A-hSOD1 mouse line. Transgenic mice expressing the high copy number human G93A-hSOD1 mutation (B6SJL-Tg[SOD1*G93A]1Gur/J; stock number 2726;first published in [29]) were originally obtained from The Jackson Laboratory (Bar Harbor, ME). The G93A-hSOD1 transgene is maintained as a hemizygous trait by breeding hemizygous males with wild-type littermate females or imported females (C57BL/6J, Harlan, The Netherlands). We modified the ALS line to contain the K108N-SEMA3A mutation by cross-breeding male G93A-hSOD1 mice (sod/-) with homozygote K108N-SEMA3A females (N/N) to generate male ALS mice heterozygote for the K108N-SEMA3A mutation (sod/-; N/-). These males were then crossed with homozygote K108N-SEMA3A females to obtain the following genotypes per litter (see Table 1 below).

**Table 1:** G93A-hSOD1 ALS mice were bred with the K108N-SEMA3A line to create ALS mice homozygote or heterozygote for the mutant SEMA3A gene.

Group	Genotype	Phenotype	Expected % residual activity of mutant SEMA3A *	Overall repulsiveness
**ALS x N/N**	G93A-hSOD1 sod/-;	ALS mouse homozygote	Two mutant alleles, each coding for protein with 15% activity	--/+
	K108N-SEMA3A N/N	for K108N-SEMA3A		
**ALS x N/-**	G93A-hSOD1 sod/-;	ALS mouse heterozygote	One mutant allele → 15% activity	+++
	K108N-SEMA3A N/-	for K108N-SEMA3A	One WT allele → full activity	
**N/N**	G93A-hSOD1 -/-;	WT mouse homozygote	Two mutant alleles, each coding for protein with 15% activity	--/+
	K108N-SEMA3A N/N	for K108N-SEMA3A		
**N/-**	G93A-hSOD1 -/-;	WT mouse heterozygote	One mutant allele → 15% activity	+++
	K108N-SEMA3A N/-	for K108N-SEMA3A	One WT allele → full activity	

* residual activity based on *in vitro* data in [28];

--/+ residual repulsive activity mediated by mutant SEMA3A protein; +++ full repulsive activity mediated by WT SEMA3A protein

The mice were maintained on a 12 h light/dark cycle with ad libitum access to food and water. Animals were housed in littermate groups and nutritional gel was given twice a week from 8 weeks of age onwards to provide easy access to food and hydration for the G93A-hSOD1 carrying animals. Over the course of the behavioral experiment, starting at 6 weeks of age, animals were weighed weekly and at least 3 times a week from 8 weeks of age to monitor weight loss for humane endpoint euthanasia (20% loss of maximum weight). When multiple weight measurements per week were recorded, these values were averaged per week in order to chart the weekly changes in weight over the course of the experiment. Only male mice were used in the behavioral experiment to account for sex differences in behavior and lifespan [30–32]. All animal care and behavioral tests were approved and carried out in compliance with the Institutional Animal Care and Use Committee of the Royal Netherlands Academy of Sciences. All research carried out with animals did comply with the current legislation and regulations in the European community (directive 2010/63/EU on protection of animals used for scientific purposes).

### Genotyping

Genomic DNA of all mice was isolated from collected ear snips by overnight (O/N) digestion at 56°C in 500μl lysis buffer pH 8.0 (100 mM Tris-HCl, 5 mM EDTA, 50 mM NaCl, 0.5% SDS) containing 1 mg/ml proteinase K. The following day, the sample was centrifuged (14,000rpm for 3 minutes) and the supernatant was transferred into a fresh tube containing 500μl isopropanol. The solution was mixed thoroughly and the precipitated DNA was pelleted by centrifugation (14,000rpm for 3 minutes). The supernatant was discarded and the pellet was dried at 56°C for 2–3 h. Subsequently, 100μl TE (10 mM Tris-HCl pH 8.0, 1 mM EDTA) buffer was added to the pellet and incubated O/N at 56°C.

#### For the behavioral experiment

The presence of the K108N-SEMA3A allele was determined as outlined below, with an additional approach to identify carriers of the G93A-hSOD1 gene. The following primers pairs were used: IL2fw 5’-CTAGGCCACAGAATTGAAAGATCT-3’; IL2bw 5’-GTAGGTGGAAATTCTAGCATCATCC-3’; SOD1fw 5’-CATCAGCCCTAATCCATCTGA-3’ and SOD1bw 5’-CGCGACTAACAATCAAAGTGA-3’. These amplify either a 236bp fragment indicative for mice carrying the mutant human G93A-hSOD1 or a 324bp fragment when mice do not carry the mutant gene [33], with the IL2 primers serving as an internal positive control.

#### For the BotoxA-induced paralysis experiment

Mice were genotyped for the K108N-SEMA3A mutation using allelic discrimination (according to ABI7300 allelic discrimination manual, Applied Biosystems) with the following primers: 808Geno-Fw—CTTACACAAGGAGAGATGAATGC; 808Geno-Rv—ACGAGAGAAGAGATCACATAGTAG and fluorescent probes: WT FAM probe—[6FAM]TGGGCTGGAAAAGATATCCTGGTAAGC[BHQ1]; Mutant JOE probe—[JOE]TGGGCTGGAAATGATATCCTGGTAAGC[BHQ1]. The ratio of red fluorescence (JOE; 6-carboxy-4',5'-dichloro-2',7'-dimethoxyfluoresceine) and green fluorescence (FAM; 6-carboxyfluorescein) determines whether the animal is a WT, a heterozygote mutant or a homozygote mutant.

### Behavioral testing

#### Rotarod

To assess overall motor coordination, an accelerating Rotarod paradigm was used (model 47600, Ugo Basile Biological Research Apparatus, Italy). Animals were placed on a rotating beam (3 cm diameter) and the latency to fall (in seconds) was measured. An arbitrary cut-off time of 180 s was chosen [34–36] during which the rotation of the beam increased from 5 to 15 rpm in the first 60 seconds and was then held constant until the end of the trial. Each animal was given three attempts and the longest latency to fall was recorded. At 4 weeks of age, the animals were allowed to familiarize with the Rotarod for three 180 s trials with the rod rotating at a constant speed of 5 rpm. Starting at 5 weeks of age, the animals were tested weekly.

#### Paw Grip Endurance

The Paw Grip Endurance (PaGE) test measures muscular strength of the limbs [37]. Each mouse was placed on the wire-lid of a conventional housing case. The lid was gently shaken to prompt the mouse to hold onto the grid before the lid was swiftly turned upside down, approximately 50 cm above the surface of soft bedding material to avoid injuries. The latency until the mouse falls of the grid was timed, with an arbitrary maximum of 90 s [35]. Each mouse was given three attempts and the longest latency to fall was recorded. Starting at 5 weeks of age, the animals were tested weekly.

### Botulinum Toxin A treatment

Isoflurane anaesthetized mice were injected unilaterally in the gastrocnemic muscle of the right hind limb with Botulinum Toxin A (dose of 0.01U/g mouse in 0.9% saline; BotoxA, Allergan, Westport, Ireland) administered via two injection sites in the muscle, with a total volume of 100ul. To create a chronic paralysis paradigm, these mice were administered with a second BotoxA dose (0.25U in 0.9% saline) 7 days later. Mice were then euthanized 7 days later (14 days after the initial BotoxA treatment; see Table 2 below).

**Table 2:** Overview of mice used in the BotoxA-induced paralysis paradigm.

Group	Genotype	Sex	Number of animals perfused after 14days of exposure to BotoxA*
**WT**	WT litter mate “-/-“	♂	4
		♀	3
**N/-**	K108N-SEMA3A heterozygote “N/-”	♂	4
		♀	5
**N/N**	K108N-SEMA3A homozygote “N/N“	♂	4
		♀	4

*BotoxA doses: day 1 dose 0.01U/g mouse; at day 7 dose 0.25U/mouse

### Tissue preparation

At 14 days post-BotoxA treatment, animals were euthanized with an overdose of Nembutal (sodium pentobarbital, Sanofi Santé) and transcardially perfused with saline followed by 4% paraformaldehyde (PFA) in 0.1M phosphate buffer (PB, pH 7.4). The right and left gastrocnemic muscles and the L3-5 portion of the spinal cord were dissected from the perfused animals and post-fixed in 4% PFA for several hours. An overnight incubation in 250mM EDTA (in 0.1M PB) to enhance antibody penetration [38] was followed by immersion in 30% sucrose (in 0.1M PB). The tissue was subsequently snap frozen in dry-ice cooled isopentane and stored at -80°C until use.

### Quantification of neuromuscular junction integrity

Immunohistochemistry was performed on longitudinal cryosections (40um thick) of the right and left gastrocnemic muscle as follows. Sections were defrosted for 30 minutes at room temperature (RT) and subsequently incubated in 50mM EDTA/0.1M PB for an additional 30 minutes at RT. Sections were rinsed in 0.1M Tris-buffered saline pH 7.4 (TBS) and blocked for 1 hour in 5% Fetal Calf Serum in TBS with 0.2% Triton-X (block-mix). Primary antibodies (from Developmental Studies Hybridoma Bank, Iowa City, IA) against neurofilament (NF-2H3, 1:250) and synaptic vesicle protein 2 (SV2, 1:250) were diluted in block-mix and added to the sections for an overnight incubation at 4°C. The following day sections were rinsed with TBS and incubated with appropriate fluorescently-labeled secondary antibodies and fluorescently-tagged bungarotoxin (FITC- or Alexa Fluor 594-labelled, Molecular Probes, 1:1000). Adjacent series were stained for muscle fiber subtype I and IIa using N2.261 (DSHB, 1:100); these sections were incubated O/N in 0.2%SSC/50% formamide at 56°C to enhance antibody penetration. Subsequently the slides were rinsed in 0.1M TBS and processed for IHC as outlined above. For quantification purposes, unstained fibers were considered as TypeIIb.

The morphology of NMJs on fibers in TypeI/IIa-positive or in unstained regions containing Type IIb-muscle fibers was scored as normal (i.e. innervated with full overlap of NF-2H3/SV2 and BTX staining) or containing sprouts (i.e. NF-2H3/SV2 positive sprouts protruding from the BTX-positive area of the NMJ). Images (z-stacks) of individual NMJs were collected using a confocal laser scanning microscope (Leica), and flattened in order to more easily visualize thin neuronal sprouts. Only NMJs positioned parallel to the imaging plane were scored. Per genotype, 100–150 NMJs were scored as sprouting or normal in TypeI/IIa or Type IIb muscle regions and at least 7 animals per genotype were analyzed.

### Statistical analysis

Behavioral data was analyzed using a repeated measures ANOVA, followed by a Bonferroni post-hoc test to identify significant differences between groups over time. A Students T-test was used to compare the sprouting capacity between two groups. Statistical significance was set at p≤0.05.

## Results

### Motor function is not altered in ALS mice expressing the K108N-SEMA3A variant

**Fig 1:**
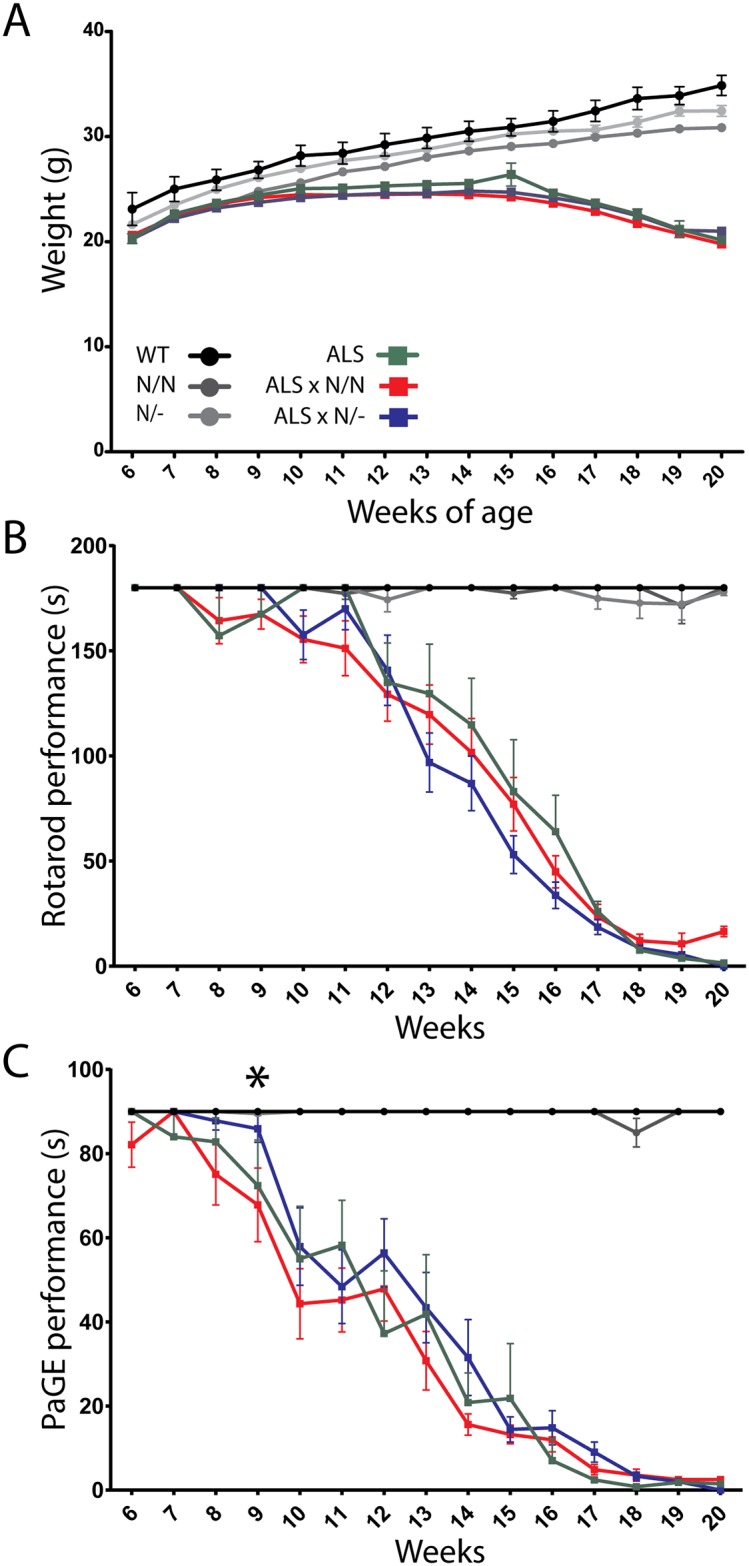
ALS mice harboring the K108N-SEMA3A gene variant continue to display a similar decline in motor function compared to “normal” ALS mice. G93A-hSOD1 mice were crossbred with K108N-SEMA3A mice to produce homozygote and heterozygote ALS mice with respect to the K108N-SEMA3A gene. The expression of K108N-SEMA3A in ALS mice did not alter ALS-induced weight loss (panel A; compare red and blue curves with green curve). From 6 weeks of age, mice were subjected to weekly Rotarod (B) and Paw Grip Endurance (PaGE; C) behavioral testing. Rotarod performance of both ALS x N/N (homozygote; red curve; panel B) and ALS x N/- (heterozygote; blue curve; panel B) shows a similar decline over the course of the 14 weeks tested, starting at approximately 11 weeks of age. The progression of decline in performance is similar to that of ALS mice harboring the WT SEMA3A gene (green curve; panel B). Similarly, for PaGE performance, ALS x N/N (red curve; panel C) and ALS x N/- (blue curve; panel C) mice show a similar decline in motor performance. However, the initial decline in performance is delayed in ALS x N/- mice compared to ALS x N/N mice (* p>0.05; week 9). Overall, neither the ALS x N/N or ALS x N/- mice show a difference in performance compared to ALS mice harboring the WT SEMA3A gene (green curve; panel C).

We hypothesized that ALS mice with diminished SEMA3A signaling would perform better in the two motor tasks compared to ALS mice expressing wild type SEMA3A. G93A-hSOD1 ALS mice were crossbred with K108N-SEMA3A mice to generate heterozygote or homozygote offspring with respect to the K108N-SEMA3A mutation (see Table 1). Over the course of the experiment, there were no differences in ALS-induced weight loss between ALS mice homozygote for the K108N-SEMA3A mutation (Fig 1*A*; ALS x N/N, red curve) and ALS mice heterozygote for the K108N-SEMA3A mutation mice (Fig 1*A*; ALS x N/-, blue curve): these animals followed the same progressive decrease in weight loss as the ALS mice expressing the wild-type SEMA3A (Fig 1A; ALS, green curve).

Between the ages of 6 weeks and 20 weeks, mice were subjected to weekly behavioral testing. Rotarod performance declines over time in a similar fashion whether the ALS mouse harbors the homozygous SEMA3A gene variant (ALS x N/N; red curve) or the heterozygous SEMA3A gene variant (ALS x N/-; blue curve) (Fig 1*A*). The decline in performance is also comparable to ALS mice expressing the WT SEMA3A gene (ALS; green curve; Fig 1*B*). In the PaGE test, ALS x N/- mice (Fig 1*C*; blue curve) tend to have a slightly better performance compared to ALS x N/N mice (Fig 1*C*; red curve), but overall the decline is similar between the two groups. Interestingly, the initial decline in PaGE performance between weeks 7–9 is not as severe in ALS x N/- mice; at week 9 these mice are performing significantly better than their ALS x N/N counterparts (* p<0.05; Fig 1*C*). Overall neither group displays a significant alteration in the decline in PaGE performance compared to ALS mice expressing the WT SEMA3A gene (Fig 1*C*; green curve). In addition, the presence of the K108N-SEMA3A mutation also does not alter ALS-induced muscle wasting or confer a survival benefit on ALS mice harboring the mutation (S1 Fig).

### The sprouting capacity of NMJs on TypeIIb muscle fibers in mice expressing the K108N-SEMA3A variant remains low after BotoxA-induced muscle paralysis

**Fig 2:**
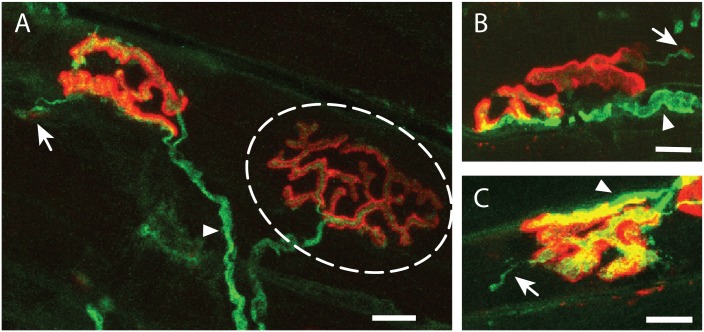
Representative immune stainings of neuronal sprouts observed at mouse gastrocnemic muscle neuromuscular junctions 14 days after BotoxA-induced paralysis. Neuromuscular junctions (NMJs) were visualized with bungarotoxin (BTX; red) and antibody staining against neurofilament and SV2 (NF-2H3/SV2; green). Thin, neuronal sprouts (arrow) are clearly distinguishable from the original, thicker motor neuron synapsing onto the endplate (arrowhead; panels A, B and C). A normal NMJ is depicted inside the dashed oval which shows a complete overlap of the BTX and NF-2H3/SV2 positive regions (panel A). A neuronal sprout (green protrusion from the NMJ on the left of panel A, and in panel B) contains the beginnings of an ectopic endplate (faint red region; arrow). A neuronal sprout which has yet to synapse on an ectopic endplate is depicted in panel C (arrow). Scale bar is 10μm.

We previously showed a close correlation between the expression of SEMA3A in TSCs and BotoxA-induced sprouting capacity of the motor neurons: motor endplates on Type I/IIa muscle fibers show abundant sprouting and an absence of SEMA3A expression in TSCs, while no sprouting is seen at TypeIIb endplates which do express SEMA3A in their TSCs [4]. To investigate whether diminished SEMA3A signaling at TypeIIb endplates results in enhanced sprouting at these end plates following BotoxA-induced denervation, the right gastrocnemic muscle was injected with BotoxA to induce paralysis and the resulting effects on sprouting at the NMJ were analyzed in TypeIIb and TypeI/IIa-muscle fiber regions. Following 14 days of BotoxA-induced paralysis, the gastrocnemic muscle was harvested, and sectioned to allow for immunostaining of neurofilament and SV2 (to visualize the motor neuron and the end terminal) and labelling of the post-synaptic acetylcholine receptors with fluorescently-labelled bungarotoxin. NMJs were scored as normal (Fig 2A; dashed oval: overlap of the green and red signals) or as containing sprouts (Fig 2A, 2B and 2C; arrows: green signal extending from red pretzel-like structure). Some ectopic junctions were identified (Fig 2A and 2C; arrows), but were not classified differently than the more common, less-mature, neuronal sprouts, given that we were interested in any neuronal growth from the main NMJ in response to BotoxA-induced paralysis (Fig 2B; arrow). The percentage of NMJs containing sprouts was determined across the different muscle fiber subtype regions (determined by immunostaining for TypeI/IIa fibers on an adjacent series of sections), and between male and female muscle.

**Fig 3:**
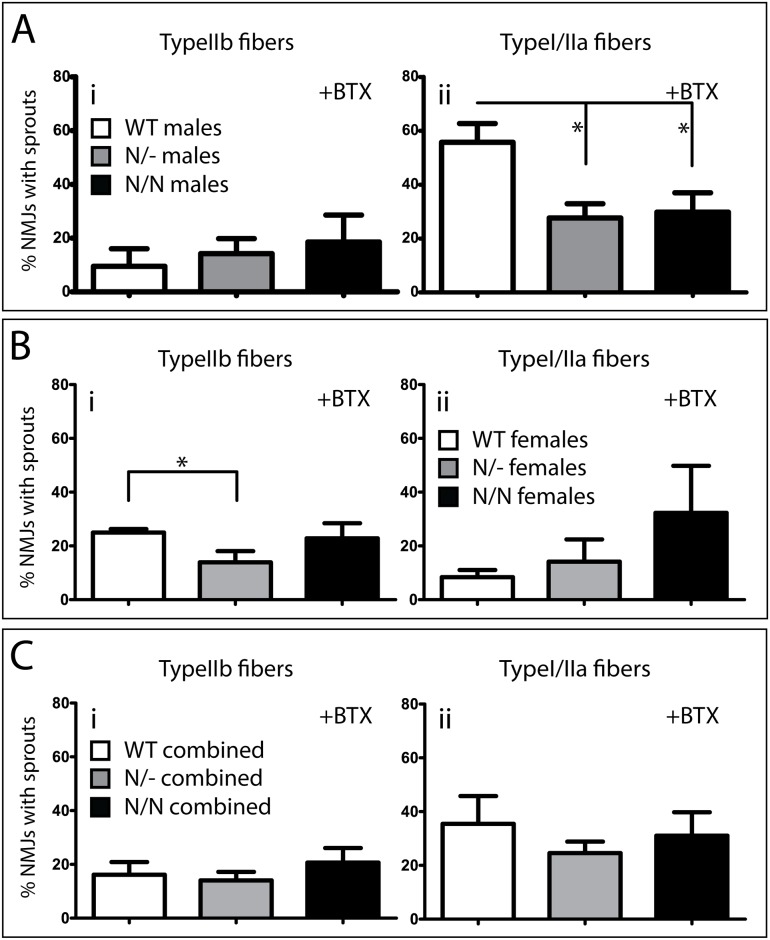
Expression of a mutant SEMA3A variant (K108N-SEMA3A) does not significantly alter the sprouting capacity at non-plastic NMJs after BotoxA-induced muscle paralysis in male mice. Neuromuscular junctions in TypeIIb and TypeI/IIa regions of wild-type (WT), K108N-SEMA3A heterozygote (N/-) and K108N-SEMA3A homotzygote (N/N) mouse gastrocnemic muscle were scored as normal or displaying sprouts 14 days after administration of BotoxA (two doses; at d0 and d7). There is a slight increase in the number of sprouts on NMJs at TypeIIb muscle fibers in heterozygote (N/-) and homozygote (N/N) males compared to WT males (Panel A; i). The number of neuronal sprouts on NMJs at TypeI/IIa muscle fibers decreases significantly in male mice expressing the SEMA3A mutant isoform (Panel A; ii. *p≤0.05). Female mice exhibit a different degree of neuronal sprouting after BotoxA administration (Panel B): WT females show a significantly higher number of NMJs with sprouts in the TypeIIb-positive region compared to heterozygote (N/-) females (Panel B; i. *p≤0.05), but not compared to the homozygote (N/N) females. In the TypeI/IIa-positive region, female mice expressing the SEMA3A mutant isoform show a non-significant increase in the number of NMJs exhibiting sprouts (Panel B; ii). In addition, WT females show a significantly higher sprouting capacity at TypeIIb fibers compared to the WT male counterparts (compare white bars in Panel B; i; and Panel A; i; respectively, p<0.05). The sprouting behavior at TypeI/IIa fibers is significantly decreased in WT females (white bar in Panel B; ii) compared to the WT male counterpart (white bar in Panel A; ii; p<0.005).

In accordance with the literature, non-plastic NMJs in the TypeIIb fiber region of male wild-type gastrocnemic muscle display a significantly lower (p = 0.0015) sprouting capacity (Fig 3Ai: white bar, 9.5%) compared to plastic NMJs in the TypeI/IIa-fiber region (Fig 3Aii: white bar, 55.7%). After BotoxA treatment, male mice heterozygote (N/- males; grey bar, 14.2%) or homozygote (N/N males; black bars, 18.5%) for the K108N-SEMA3A gene show a slight, but non-significant, increase in the sprouting capacity at NMJs in the Type IIb muscle fiber region compared to WT males (Fig 3Ai: compare grey and black bars with white bar, respectively). On the other hand, the presence of the K108N-SEMA3A variant significantly decreases the sprouting capacity at NMJs on TypeI/IIa muscle fibers in heterozygote (p = 0.01; N/- males; grey bar, 27.6%) or homozygote (p = 0.02, N/N males; black bar, 29.8%) males compared to WT males (Fig 3Aii: compare grey and black bars with white bar, respectively).

Female mice heterozygote (Fig 3Bi: grey bar, 13.9%) or homozygote (Fig 3Bi: black bar, 22.8%) for the K108N-SEMA3A gene show a similar sprouting capacity in the TypeIIb muscle fiber region to males of the same genotype (compare grey and black bars in Fig 3Ai with 3Bi). However, contrary to WT males, WT females display a significantly higher (p = 0.006) sprouting capacity at NMJs in the usually non-plastic TypeIIb region (Fig 3Bi: white bar, 25%) compared to those in the Type I/IIa region (Fig 3Bii: white bar, 8.4%).

The difference in sprouting capacity between males and females was also analyzed. After BotoxA treatment, there are significantly more sprouts at NMJs in the TypeIIb region in WT female muscle compared to WT male muscle (Fig 3Ai: white bar, compared to Fig 3Bi: white bar. p = 0.048). Heterozygote and homozygote animals do not show a sex difference in sprouting capacity at the NMJs of TypeIIb muscle fibers (grey and black bars, compare Fig 3A and 3B). In untreated muscle, the neuronal sprouting capacity at NMJs in the TypeIIb region between male and female groups does not differ significantly; it remains low (≤12%; data not shown). When the sprouting percentages of both male and female groups are pooled (Fig 3C) there are generally more sprouts (after BotoxA administration) at NMJs in the TypeI/IIa region compared to the TypeIIb region (Fig 3C; compare genotype groups in i and ii; with the heterozygote group (grey bar) showing significantly more sprouting in the TypeI/IIa region, p = 0.03). However, there are no significant differences in sprouting capacity when comparing the sprouting differences across genotypes within the TypeIIb region (Fig 3Ci) or the TypeI/IIa region (Fig 3Cii). The numerical data depicted in Fig 3 is provided in S1 Table. A complete overview of significant differences in sprouting capacity across fiber type, sex or genotype is tabulated in S2 Table.

## Discussion

In the current study we used the K108N-SEMA3A mutant mouse to directly study the influence of SEMA3A on the ALS phenotype and NMJ morphology in an effort to specifically impair SEMA3A function without affecting the ability of NRP1 to interact with its additional ligands. The K108N-SEMA3A mutant retains the ability to bind to its receptor, NRP1, but the interaction between K108N-SEMA3A-NRP1 and the signal-transducing component of the holoreceptor, Plexin A, is impaired. The mutation in SEMA3A creates a protein with diminished signaling properties [approximately 85% less potent than WT SEMA3A [28]]. It is important to note, however, that the K108N-SEMA3A protein is capable of eliciting a full repulsive effect on neurons in culture when applied at higher concentrations [28]. Keeping this in mind, the K108N-SEMA3A mouse therefore is a “partial” SEMA3A knockout. Previous attempts to create a true SEMA3A knockout to study its role in adulthood failed. Behar and colleagues described that ~70% of SEMA3A homozygote knockouts die within the first 3 days [10], and Taniguichi and colleagues describe their homozygote knockouts as “largely viable” but give no indication to the numbers surviving into adulthood [39]. The K108N-SEMA3A mutant mice display a similar developmental neuronal phenotype to the SEMA3A-null mice, i.e. aberrant defasciculation of cranial and spinal nerves, and overgrowth of ulnar and radial nerves into their target areas, but importantly, are described as “homozygote viable”compared to the SEMA3A-null mice [28]. The K108N-SEMA3A mouse (homozygote and heterozygote) displays a normal behavioral phenotype compared to wild-type mice and remains fertile throughout adulthood (our observations). The discrepancy in viability between the traditional SEMA3A knockouts and the K108N-SEMA3A line may be explainable by the residual SEMA3A function attributed to the mutant K108N-SEMA3A.

We cross-bred the K108N-SEMA3A mouse with the G93A-hSOD1 ALS-mouse to create an ALS mouse which is either heterozygote (N/-) or homozygote (N/N) in terms of the K108N-SEMA3A mutation. The G93A-hSOD1 ALS mouse represents a model for a relatively rare genetic form of ALS in human patients, but we chose this mouse model because it is one of the best characterized ALS-mouse models. The behavioral data indicate that the presence of a SEMA3A mutant isoform with 85% diminished signaling potency does not alter the ALS-induced decline in performance on Rotarod or PaGE behavioral tests. These results can be interpreted in multiple ways. First, this may endorse the idea that factors other than SEMA3A have a much greater role in the motor decline observed in ALS [40]. Secondly, in ALS mice expressing the attenuated form of SEMA3A the relatively low levels of SEMA3A-mediated repulsion may be still sufficient to induce a motor-deficit phenotype. There is no behavioral difference between ALS mice who express one or two mutant alleles, suggesting that the small amount of remaining repulsive activity of the SEMA3A mutant protein in the ALS mouse carrying the two mutant alleles may be sufficient to maintain a similar behavioral decline seen in ALS mice carrying one mutant allele or those expressing WT SEMA3A. This is consistent with the notion that SEMA3A is a very potent repulsive factor. Picomolar quantities of the protein are sufficient to induce full growth cone collapse *in vitro* [41]. The presence of the K108N-SEMA3A mutation also does not alter ALS-induced muscle wasting or confer a survival benefit on ALS mice harboring the mutation (S1 Fig).

The observations reported here appear to be in contrast with a recent study by Venkova and colleagues (26). In that study, peripheral administration of monoclonal antibodies that specifically interfere with SEMA3A binding to NRP1 in the same ALS mouse strain used by us improved motor function and significantly increased life span in the ALS-mice. The antibody was most effective when administered during the early stages of the disease process. An important difference between this study and our study is that the antibody treatment *acutely* interferes with SEMA3A-NRP1 signaling in adult ALS-mice while cross-breeding of ALS mice with K108N-SEMA3A mutant mice results in animals with a *chronic* and partial defect in SEMA3A signaling. Persistent chronic changes in SEMA3A-NRP1 signaling that occur throughout development may result in compensatory processes that could mask the role of SEMA3A in the ALS disease process. These apparently contradictory findings warrant further research aiming to better understand the role of Sema3A-NRP1 signaling in the ALS disease process.

NRP1 is also a receptor for VEGF [42] and Transforming Growth Factor (TGF)-beta1 [43] and mediates Platelet-derived Growth Factor (PDGF) signaling [44]. Although Venkova and colleagues used an antibody that was designed to specifically interfere with the interaction of SEMA3A with NRP1 [26] this does not exclude the possibility that this antibody has effects on NRP1 signaling capabilities non related to SEMA3A.

It is possible that although no behavioral improvement was detected in ALS mice expressing the K108N-SEMA3A mutation, the mutant protein has an effect on the plasticity of NMJs themselves without this translating into a measurable functional outcome. We sought to identify the influence of mutant SEMA3A in NMJ plasticity by using a BotoxA-induced paralysis paradigm in the K108N-SEMA3A mouse. NMJ plasticity is measurable by analyzing the extent of motor neuron sprouting in response to paralysis of the target muscle with muscle fiber subtypes displaying different sprouting capacities of their synapses. Motor axons on slow muscle fibers exhibit extensive sprouting after BotoxA-treatment while in contrast motor axons in fast-fatigable muscle fibers display limited sprouting. The fast-fatigable, TypeIIb fibers which are first to be denervated in ALS are also the type that show limited sprouting after denervation or injury [5].

There are several studies that have illustrated that BotoxA-induced sprouting capacity of motor neurons on fast-fatigable (TypeIIb) type muscle fibers is generally lower than on the slow (TypeI) and fast fatigue-resistant (TypeIIa) type fiber counterparts [4,5,45]. In accordance with this, the WT male mice in our study display an increased level of sprouting at NMJs in the TypeI/IIa (“plastic”) region compared to the TypeIIb (“non-plastic”) region after BotoxA-induced muscle paralysis. However, we show that the sprouting capacity of NMJs at TypeIIb fibers is not increased by the presence of the mutant SEMA3A protein, i.e. the plasticity of NMJs in the K108N-SEMA3A heterozygote or homozygote male mouse is not significantly different compared to the NMJs of TypeIIb fibers in WT male mice. This is consistent with the finding that the mutant SEMA3A protein retains some repulsive activity. This residual repulsive signal may be enough to keep the sprouting capacity of motor neurons on TypeIIb fibers on a par with the WT males. Alternatively, this data might indicate that the selective induction of SEMA3A at TypeIIb endplates may not have a role in restricting sprouting at these NMJs.

Surprisingly, after BotoxA treatment the sprouting capacity at NMJs of TypeI/IIa fibers is significantly decreased in both N/- and N/N male animals compared to the same fiber region in BotoxA-treated WT males. As a result, the sprouting capacity between the fiber subtypes (TypeI/IIa versus TypeIIb) is not significantly different in N/- or N/N mice. Thus, one could say that the expression of the mutant form of SEMA3A (with diminished signaling capacity) removes the differential sprouting capacity between the fiber subtypes in transgenic males. The biological meaning of this remains unclear, especially because there were no overt differences in the degree of paralysis observed in WT and N/N or N/- transgenic mice after BotoxA treatment (our non-quantitative observations).

Female mice, on the other hand, displayed an interesting, and unexpected, switch in terms of the baseline sprouting capacity in the TypeIIb (“non-plastic”) region compared to the TypeI/IIa (“plastic”) region: WT females display a significantly higher level of sprouting at NMJs in the TypeIIb region compared to the TypeI/IIa region after BotoxA treatment. These results point to a very clear sex-difference in sprouting capacity, which has not been described in the literature before. The majority of studies using a BotoxA-induced denervation paradigm to study NMJ plasticity use male rodents (or do not specify the sex of animals used) [4,5,45–47], and thus do not allow a direct comparisons with our data in female mice. Nevertheless, there are several papers that specifically use female mice, and upon BotoxA-induced paralysis describe an increase in motor neuron sprouting on fast-twitch muscle fibers [48–50]. However, these studies fail to distinguish between the two fast-twitch muscle fiber subtypes, TypeIIa and TypeIIb, making it possible that the sprouting behavior they describe is due to sprouting at the TypeIIa (“plastic”) fibers rather than at the TypeIIb (“non-plastic”) fibers, which would be consistent with data obtained from male-only or mixed-sex experimental groups.

There are several possibilities as to why we observe a sex dimorphism in terms of sprouting capacity on usually “non-plastic” (TypeIIb) muscle fibers in female muscle. Firstly, as mentioned above, the literature that explicitly describes sprouting in female fast-twitch muscle does not distinguish between TypeIIa (“plastic”) or TypeIIb (“non-plastic”) [48–50], and although the increase in sprouting may be solely due to sprouting on TypeIIa fibers in these muscles, we cannot rule out that female TypeIIb fibers also support sprouting after BotoxA-paralysis (an idea which is supported by our data). Secondly, there are many documented sex differences in skeletal muscle physiology in development and in response to injury or disease [51,52], and this itself may alter the sprouting responses of the motor neuron. Hormones have been linked to the regulation of synapse elimination during (post-natal) development [53,54], supporting the idea of sex dimorphism at the NMJ itself [55]. The fiber composition of skeletal muscle is also influenced by sex; hormones play a role in the development of sex dimorphic skeletal muscle and can also influence the fiber type distribution in individual muscles [51,56–58], for e.g. some male muscles may have relatively more slow, Type I fibers than the same muscle in females, which allows for increased endurance function, thus generating muscles that are capable of sustaining contractile strength. Muscle fibers are also capable of transitioning between subtypes (creating so-called hybrid fibers) in response to injury- or disease-induced changes in synaptic input [59–61] as a compensatory mechanism to maintain muscle function, albeit an altered version of the muscles’ original force and twitch abilities. This mechanism is a dynamic process, and uses a “nearest-neighbor method” to determine which subtype the transitioning fiber will become [60]. Due to these sex-specific differences in skeletal muscle, one could expect a differential response to denervation, since their regenerative capacity and fiber subtype composition is already different given the hormonal (and thus differential) regulation between males and females. As such, it is no surprise to see that the motor neuron sprouting behavior in males and females is so different after BotoxA-induced paralysis of the skeletal muscle.

Interestingly, ALS shows a sex bias; males are usually more affected than females (incidence ratio of approximately 2:1), with onset of the disease occurring much later in females [31,62,63]. However, once a motor unit is deteriorating, the progressive nature of this loss is unaffected by sex [64]. Nevertheless, a recent study determined that sex differences do already exist at the level of NMJ function in the presymptomatic stages in ALS mice, with female ALS mice displaying no change in the readily releasable pool (RRP) of synaptic vesicles compared to WT females, whereas ALS males display a significant decrease in RRP [55]. This is consistent with the finding that enhanced neuromuscular transmission (and therefore reduced RRP) in males is also measurable in the early stages of the disease [65,66]. Hormonal differences are thought to be one of the major reasons behind the sex dimorphism in ALS; female reproductive hormones have been shown to have a neuroprotective effect on motor neurons [67,68]. Growth hormone elevation occurs upon symptom onset in ALS and it is thought to be a compensatory mechanism to delay muscle atrophy by stimulating IGF-1 production, a growth factor known to stimulate reinnervation of the muscle [69]. Interestingly, females secrete, via estrogen-dependent mechanisms, far higher levels of growth hormone compared to males [70] which may be an additional mechanism influencing the sex specificity of ALS. Recently, a direct link between the SEMA3A-NRP1 signaling pathway and the regulation of female reproductive hormones was discovered [71,72]. In this study, Giacobini and colleagues show that SEMA3A released from brain endothelial cells is regulated by the ovarian cycle and promotes axonal sprouting in hypothalamic neurons to stimulate the release of fertility hormones [71]. Fertility hormones have also been shown to influence the PNS; progesterone can enhance Schwann cell proliferation and morphology, and stimulate functions associated with myelination [73], an important feature in peripheral nerve health and function.

The sex differences we see in our data on motor neuron sprouting after BotoxA-induced paralysis can potentially be linked to the sex dimorphism seen in ALS (see Table 3 below). Our data illustrate that (in accordance with the literature) motor neurons synapsing on TypeIIb muscle fibers show limited plasticity after BotoxA-induced paralysis of *male* gastrocnemic muscle. These synapses are the first to degenerate in ALS [5] presumably because they are unable to adapt (i.e. sprout) in response to ALS-induced changes at the NMJ, such as re-expression of SEMA3A in TSCs at these junctions in ALS [and after BotoxA-induced paralysis; [4]]. On the other hand, motor neurons synapsing on TypeI or TypeIIa muscle fibers demonstrate a robust sprouting phenotype after BotoxA-induced paralysis, and are the least vulnerable to denervation over the course of the ALS disease progression. In females, however, who are less likely to develop ALS (incidence ratio is 2:1 male to female), the sprouting behavior of motor neurons after BotoxA-induced paralysis is altered compared to males, and may represent an additional sex dimorphic difference in motor neurons: Naumenko and colleagues showed that sex differences exist at the pre- (and post-)synaptic level which affect the onset and development of ALS [55]. Our data indicates that motor neurons synapsing on TypeIIb muscle fibers have an increased sprouting capacity in females compared to similar NMJs in males (Fig 3, p<0.05). Because these junctions are more plastic, they may be able to remain functional for longer, which, in the ALS disease scenario, may translate to female ALS muscle remaining innervated for longer compared to male ALS muscle.

**Table 3:** Sex differences observed in motor neuron sprouting behavior and risk for ALS.

	Plasticity after BotoxA treatment		Risk of ALS
	*IIb fibers*	*I/IIa fibers*	
Males	---	+++	+++
Females	-/+	-/+	-/+

---low sprouting capacity; +++ high sprouting capacity / high risk of ALS; -/+ low sprouting capacity / low risk of ALS

Our data on the sex dimorphic sprouting behavior of WT motor neurons synapsing on TypeIIb or TypeI/IIa muscle fibers may also illustrates a potential interdependency between muscle fiber subtypes in their sprouting capacity. In other words, when sprouting does *not* occur at NMJs on TypeIIb fibers upon denervation (as is the case in males), the response of motor neurons on TypeI/IIa fibers is to increase their sprouting behavior, perhaps as a compensatory effect to try to maintain appropriate muscle function (albeit, with a decrease in force capabilities over time as the saved fibers may turn into slow-twitch fibers). In contrast, females show a slight, yet significant, increase in sprouting of NMJs at TypeIIb muscle fiber compared to males upon denervation of the target muscle. This coincides with a low sprouting response at the NMJs at TypeI/IIa, which is consistent with the idea that these junctions do not need to compensate by increasing their sprouting behavior because the TypeIIb fibers continue to be innervated by the correct motor neuron subtype (and therefore the muscle, as a whole, retains its original force twitch capacity for longer). This line of reasoning may also be one aspect as to why the onset and progression of amyotrophic lateral sclerosis is delayed in females, i.e. the skeletal muscle remains appropriately innervated for longer.

In conclusion, the presence of a mutant isoform of SEMA3A, with decreased signaling capacity, does not alter the amyotrophic lateral sclerosis related decrease in motor function. This indicates that SEMA3A signaling may be of little importance as a factor that compromises the integrity of NMJs in ALS. This is in contrast with a recent study showing that administration of antibodies that interfere with SEMA3A binding to NRP1 in ALS-mice improved motor function and increased life span in these mice [26]. The antibody treatment *acutely* interferes with SEMA3A-NRP1 signaling in adult ALS-mice while cross-breeding of ALS-mice with K108N-SEMA3A mutant mice results in animals with a *chronic* partial defect in SEMA3A signaling. Persistent chronic changes in SEMA3A-NRP1 signaling may result in compensatory processes that could mask the role of SEMA3A in the ALS disease process. In line with this reasoning, we also cannot exclude the possibility that the residual chemorepulsive activity retained by the mutant protein is sufficient to negatively influence the stability of the NMJ. The best approach for studying the influence of SEMA3A-NRP1 signaling at the NMJ in ALS would be to completely ablate the SEMA3A gene specifically in TSCs and only in late post-natal life. Acutely targeting the SEMA3A–NRP1 signaling pathway during late post-natal life, would avoid the potential compensatory effects that may occur when interfering with guidance pathways important during development.

## Supporting Information

S1 Fig G93A-hSOD1 ALS mice harboring the K108N-SEMA3A gene variant do not survive longer than G93A-hSOD1 ALS mice expressing the WT SEMA3A gene (compare green line [ALS] with red and blue lines [ALS mice expressing the mutant form of SEMA3A]. (AI)

S1 Table Overview of % NMJs with sprouts 14 days after BTX treatment (with n and SEM for each group). (XLSX)

S2 Table Student T-test p-values when comparing % NMJ with sprouts 14 days after BotoxA treatment. (XLSX)
